# Comparison of visual outcomes between therapy choices and subtypes of polypoidal choroidal vasculopathy (PCV) in Taiwan: a real-world study

**DOI:** 10.1038/s41598-020-80731-1

**Published:** 2021-01-11

**Authors:** Ling Yeung, Chi-Chun Lai, San-Ni Chen, Cheng-Kuo Cheng, Chung-May Yang, Yi-Ting Hsieh, Arslan Tsai, Chang-Hao Yang

**Affiliations:** 1 Department of Ophthalmology, Chang Gung Memorial Hospital, Keelung, Keelung City, Taiwan; 2 Department of Ophthalmology, Chang Gung Memorial Hospital, Linkou, Taoyuan City, Taiwan; 3 Department of Ophthalmology, Changhua Christian Hospital, Changhua City, Taiwan; 4 Department of Ophthalmology, Shin Kong Wu Ho-Su Memorial Hospital, Shilin District, Taipei City, Taiwan; 5 Clinical Development and Medical Affairs, Novartis Taiwan, Taipei City, Taiwan; 6 Department of Ophthalmology, School of Medicine, National Taiwan University Hospital, No. 7, Zhongshan South Road, Zhongzheng District, Taipei City, 100 Taiwan

**Keywords:** Eye manifestations, Outcomes research

## Abstract

Polypoidal choroidal vasculopathy (PCV) is a distinctive type of neovascular age-related macular degeneration prevalent in many Asian countries. However, there is still some controversy in how the subtypes of PCV are classified. This post-hoc study redefined the branching vascular network (BVN) and PCV subtypes through retrospective review of indocyanine green angiography (ICGA) and fluorescein angiography images from two observational studies (RENOWNED/REAL). Of the visual outcomes for each angiographic subtype and treatment pattern investigated, BVN was identified in 56.3% of PCV patients. The proportions and features of the re-defined PCV subtypes were 43.8%, 10.4%, and 45.8% for subtype A (without distinctive features of BVN), B (with BVN but no leakage), and C (with BVN and leakage), respectively. Subtype A had better visual outcomes when compared to subtype C. This possibly resulted from a better baseline visual acuity in subtype A. Moreover, combination therapy [photodynamic therapy plus anti-vascular endothelial growth factor (VEGF)] may lead to better visual improvement than mono-anti-VEGF treatment alone. This study provides the prevalence of PCV subtypes in Taiwan and may serve as a reference for PCV treatment strategies in a real-world setting, especially for the combination therapy and patients without distinctive features of BVN.

## Introduction

Polypoidal choroidal vasculopathy (PCV), which was first named by Yannuzzi et al.^1^, is a distinctive form of neovascular age-related macular degeneration (nAMD). It is characterized by the presence of polyps and branching vascular networks (BVNs)^2^. The prevalence of PCV differs between western (8–13%) and Asian countries (20–60%)^2–10^.

The standard treatment options for PCV include photodynamic therapy (PDT), mono-anti-vascular endothelial growth factor (VEGF) therapy, and various combination regimens. However, the therapeutic efficacy of these treatments is inconsistent^3^. PDT results in a high polyp regression rate, but is less effective against BVN^11^, whereas mono-anti-VEGF therapy leads to favorable visual outcomes, but shows less sustainable effects on polyp regression^12,13^. The EVEREST II clinical study reported that PDT plus anti-VEGF was superior to mono-anti-VEGF therapy for the improvement of visual acuity and the complete polyp regression^14–16^. However, previous researchers also found that PCV patients with different characteristics using ICGA might result in various clinical outcomes^17^, which were consistent with the findings in the EVEREST study Report 5^18^.

Indocyanine green angiography (ICGA) is the standard diagnostic tool for detecting polyps and BVN in PCV^19,20^. Several studies have demonstrated that PCV can be classified into different subtypes based on angiographic and optical coherence tomography findings^21–25^. Yuzawa et al. categorized patients into polypoidal choroidal neovascularization (CNV) and typical PCV^25,26^. Tan et al. classified patients with PCV into Type A, B, and C in accordance with the presence of interconnecting channels (Type A) or BVN (Type B and C); Type B has no leakage, while Type C has leakage visualized by fluorescein angiography (FA)^17^. However, Tan et al. did not mention why no Type A patients had FA leakage in their cohort^18^.

Because of the diverse classifications of PCV subtypes, this study aimed to redefine each subtype and provide a reference for the diagnosis, therapy, and prognosis of PCV. We defined the diagnostic criteria for BVN adopted in clinical settings^27^ by encompassing a broad range of BVN concepts, from Yuzawa’s feeder vessels^25,26^ to Inoue’s morphology^21^. Patients without distinct BVN features detected by ICGA, regardless of the status of leakage, were defined as subtype A, which differs from Tan’s Type A^18^. Patients with BVN observed by ICGA with or without FA leakage were then defined as subtypes B and C, respectively.

The data for this retrospective post-hoc study were from two multicenter, open-label, single-arm, observational studies for the use of ranibizumab in nAMD patients (RENOWNED/REAL^28,29^) and were re-analyzed to explore (1) the proportion of PCV subtypes in Taiwan and (2) to investigate the treatment outcomes of different PCV subtypes.

## Results

Table 1 shows the demographic data and baseline characteristics of the overall study population, as well as PCV subtypes. For the 67 PCV patients, the mean age was 68.9 years old with males accounting for a higher proportion of the population (64.2%). On average, patients had 3.2 polyps, with an average greatest linear dimension (GLD) of 348.5 μm. The GLD of whole lesions averaged 3194.3 μm and 46.8% of patients had cluster-type polyps.

**Table 1:** Demographic and clinical characteristics of the overall study population and PCV subtypes.

	PCV patients, n = 67	Subtype A n = 21 (43.8%)^$^	Subtype B n = 5 (10.4%)^$^	Subtype C n = 22 (45.8%)^$^	P-value^†^
**Age (years)**					0.8866
Mean ± SD	68.9 ± 11.04	66.3 ± 9.91	65.0 ± 7.11	67.3 ± 11.18	
**Gender, n (%)**					0.2830
Male	43 (64.2)	11 (52.4)	4 (80.0)	16 (72.7)	
**Baseline BCVA (letter)**					0.3939
Mean ± SD	50.8 ± 14.91	59.5 ± 12.06	50.4 ± 16.59	54.8 ± 14.24	
**Number of polyps**					0.7350
Mean ± SD	3.2 ± 2.56	3.0 ± 2.43	4.0 ± 2.45	3.2 ± 2.68	
**GLD of the largest polyp (μm)**					0.2795
Mean ± SD	348.5 ± 136.91	356.3 ± 151.63	259.2 ± 101.06	363.8 ± 118.07	
**GLD of the whole lesion (μm)**					0.9384
Mean ± SD	3194.3 ± 1672.82	3203.7 ± 1687.13	3023.8 ± 1693.84	3307.6 ± 1657.24	
**Had a cluster of polyps, n (%)**	22/47^#^ (46.8)	11 (52.4)	4 (80.0)	7 (31.8)	0.1078
Had BVN, n (%)	27/48^$^ (56.3)	NA	5 (100)	27 (100)	NA
Had SRF, n (%)	50 (74.6)	17/19 (89.5)	4 (80.0)	18 (81.8)	0.7548
Prior treated patients, n (%)	37 (55.2)	12 (57.1)	2 (40.0)	12 (54.5)	0.7865
**Treatment choice during the study period, n (%)**					0.9885
Combination therapy	24 (35.8)	9 (42.9)	2 (40.0)	9 (40.9)	
Mono-anti-VEGF therapy	43 (64.2)	12 (57.1)	3 (60.0)	13 (59.1)	
**Number of anti-VEGF injections**					0.1635
Mean ± SD	3.9 ± 2.08	4.2 ± 2.50	5.2 ± 2.59	3.3 ± 1.67	
**Number of PDT therapy**					0.4499
Mean ± SD	1.1 ± 0.31	1.2 ± 0.44	1.0 ± 0.00	1.0 ± 0.00	

*PCV* polypoidal choroidal vasculopathy, *SD* standard deviation, *GLD* greatest linear dimension, *NHI* national health insurance, *VEGF* vascular endothelial growth factor, *SRF* subretinal fluid, *BVN* branching vascular network, *NA* not applicable, *PDT* photodynamic therapy.

^#^Only 47 patients with polyps were recorded.

^$^Patients whose data were not gradable were not included in the calculation of PCV subtype distribution (not gradable: n = 19).

^†^One-way ANOVA or Chi-square tests were applied for continual or categorical variables to compare differences between subtypes.

Among the 67 PCV patients, 48 had evaluable ICGA/FA images pre-defined as prior to any exposure to PDT whereas 19 were not gradable. This resulted in 21 (43.8%) classified as subtype A, 5 (10.4%) classified as subtype B, and 22 (45.8%) classified as subtype C. Twenty-seven (56.3%) patients had BVN. Overall, there were no significant differences in demographic data between the three subtypes. Combination therapy was provided to 40.0–42.9% of the patients. Prior to participating in the RENOWNED and REAL studies, anti-VEGF injections were given to 55.2% of the patients. The number of anti-VEGF injections were 3.3–5.2. The therapy choices were well balanced among the three subtypes. PCV subtypes were not associated with the treatment pattern (i.e., combination therapy or mono-anti-VEGF therapy, p = 0.8739) or number of injections (p = 0.1620).

### Effect of therapy choice on visual and anatomic outcomes for PCV patients

**Figure 1:**
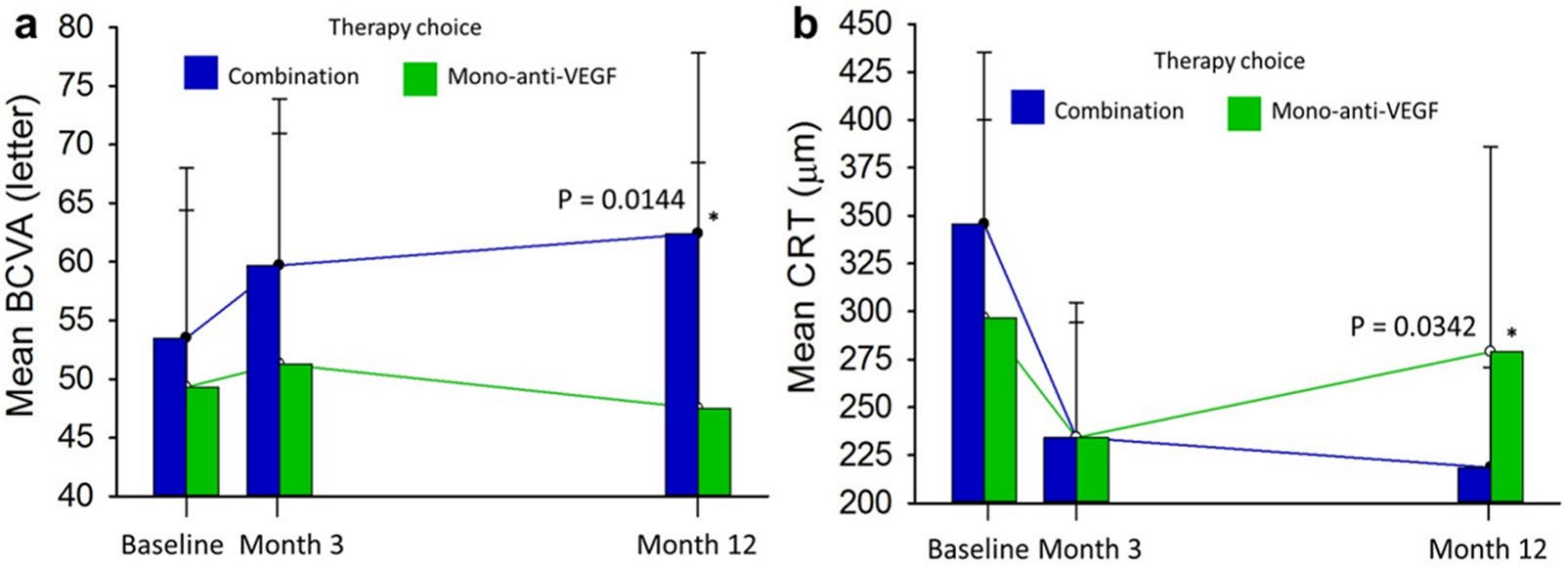
Visual outcomes by therapy choice during the 12-month treatment. (**a**) Mean BCVA. (**b**) Mean CRT. Blue line, combination therapy; green line, mono-anti-VEGF therapy. *BCVA* best-corrected visual acuity, *CRT* central retina thickness. Asterisks indicate significant differences.

The mean best-corrected visual acuity (BCVA) and CRT values compared between therapy choices are illustrated in Fig. 1. Combination therapy had better BCVA and CRT outcomes than the mono-anti-VEGF therapy at 12 months. Compared to mono-anti-VEGF therapy, an upward trend in BCVA was observed with combination therapy (Fig. 1a, and a downward trend in CRT was detected Fig. 1b).

For considering the effects of baseline levels and therapy choices, ANCOVA was used to analyze the least square (LS) mean changes (Supplementary Table S1). The LS mean change in BCVA for combination therapy was significantly higher than for mono-VEGF therapy (p = 0.0047). The LS mean reduction in CRT for combination therapy was also significantly greater than the mono-VEGF therapy (p = 0.0097).

### Odds ratio analysis of PCV patients’ visual outcomes

We also analyzed the odds ratios (OR) of sustained or improved BCVA adjusted for PCV subtypes and therapy choices (Table 2). The combination therapy was significantly associated with higher odds of improved BCVA compared to the mono-anti-VEGF therapy (OR: 5.310; 95% CI 1.044–27.012; p = 0.0442). Likewise, the combination therapy might be more likely to sustain BCVA compared to the mono-anti-VEGF therapy, despite having no significant difference (OR: 4.536; 95% CI 0.699–29.455; p = 0.1131). PCV subtypes seemed to not be associated with the response of BCVA, either in the comparisons between subtypes A and B or between A and C (both p > 0.05).

**Table 2:** Odds ratios for BCVA changes in different PCV subtypes and therapy choices.

	Odds ratio	95% CI	P-value^$^
**Sustained BCVA**			
Combination therapy vs. mono-anti-VEGF therapy	4.536	0.699, 29.455	0.1131
PCV subtype B vs. PCV subtype A	72,135	0.000, > 9999	0.9627
PCV subtype C vs. PCV subtype A	0.379	0.065, 2.215	0.2817
**Improved BCVA**			
Combination therapy vs. mono-anti-VEGF therapy	5.310	1.044, 27.012	0.0442*
PCV subtype B vs. PCV subtype A	1.917	0.131, 28.030	0.6345
PCV subtype C vs. PCV subtype A	0.612	0.117, 3.217	0.5622

*CI* confidence interval, *BCVA* best-corrected visual acuity, *PCV* polypoidal choroidal vasculopathy, *VEGF* vascular endothelial growth factor.

1. Loss ≤ 4 letters of BCVA at Month 12 was defined as sustained BCVA.

2. Increase ≥ 5 letters of BCVA at Month 12 was defined as improved BCVA.

^$^Logistic regression model was applied.

*P-value < 0.05 considered as a significant difference.

### Effect of PCV subtypes on visual and anatomic outcomes

**Figure 2:**
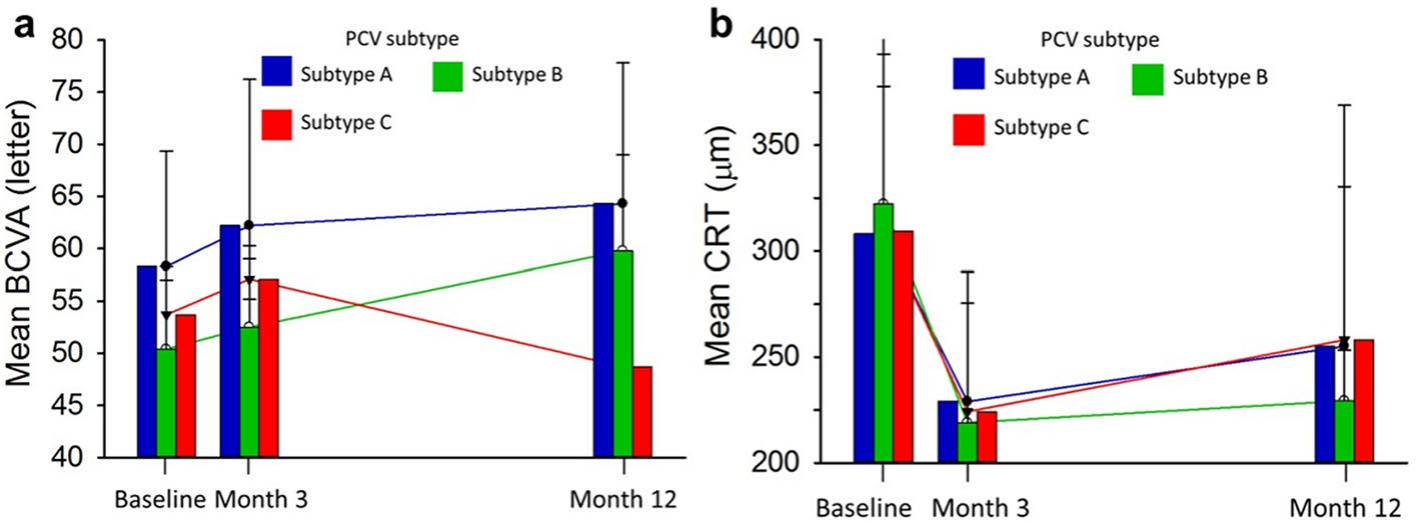
Visual outcomes by PCV subtype during the 12-month treatment. (**a**) Mean BCVA. (**b**) Mean CRT. Blue line, PCV subtype A; green line, PCV subtype B; red line, PCV subtype C. *BCVA* best-corrected visual acuity, *BVN* branching vascular network, *CRT* central retina thickness.

Figure 2 displays the visual outcomes by PCV subtypes. Overall, the mean BCVA and CRT were not significantly different between subtypes A, B, and C during the 12-month treatment. The final mean BCVA was different between subtype A (64.3 letters), B (59.8 letters), and C (48.7 letters), with borderline significance (p = 0.0839).

We also found that baseline BCVA seemed to be associated with changes in BCVA at a borderline significance (p = 0.0802; data not shown). In this respect, we adjusted the visual outcomes between PCV subtypes to account for the real-world variation in the regression model using MI. After adjustment, a significant difference in mean BCVA at month 12 was observed (p = 0.0381), which was potentially related to the baseline BCVA as a covariate and the fact that subtype A numerically has the highest baseline BCVA of 58.9 letters (Supplementary Table S2).

### Correlation analysis

The adjusted BCVA changes for each PCV subtype are tabulated in Table 3. We found that baseline BCVA (p = 0.0472) and therapy choices (p = 0.0133) were significantly correlated with the changes in BCVA. However, there were no significant differences between PCV subtypes. Patients of subtypes A or B showed improved BCVA (LS mean change in subtype A and B were 8.6 and 10.7 letters, respectively), but those of subtype C showed reduced BCVA (− 2.0 letters). This study also explored other potential predictors of treatment outcomes for PCV patients (Supplementary Table S3). Baseline BCVA (p = 0.0156) might affect changes of BCVA. In terms of anatomic outcomes, baseline CRT (p = 0.0210) and polyps found in the subfoveal location (p = 0.0062) might also impact the reduction of CRT.

**Table 3:** Adjusted changes in BCVA by PCV subtypes.

			P-value^$^		
**Covariate for BCVA changes**					
Baseline BCVA			0.0472*		
Age			0.1875		
Therapy choices			0.0133*		
Prior anti-VEGF treatment			0.3535		
SRF at Month 3			0.5112		

	n	LS mean change ± SD at 12 months		Difference between subtypes	P-value^#^
**BCVA changes (letter)**					
Subtype A	19	8.6 ± 4.56		A vs. B = − 2.1 ± 10.37	> 0.9999
Subtype B	3	10.7 ± 9.07		B vs. C = 12.7 ± 10.04	0.6546
Subtype C	19	− 2.0 ± 4.39		A vs. C = 10.6 ± 6.50	0.3539

*SD* standard deviation, *LS* least square, *VEGF* vascular endothelial growth factor, *SRF* subretinal fluid, *BCVA* best-corrected visual acuity.

^#^ANCOVA was applied for the LS mean changes by considering the effects of baseline BCVA and all covariates (age, therapy choices, prior anti-VEGF treatment, and presence of SRF at 3 months).

## Discussion

This post-hoc study was conducted to investigate the proportion of PCV subtypes in Taiwan and the treatment outcomes for each subtype following a redefined classification according to the presence of BVN from ICGA and leakage from FA. Our ICGA images showed that around 56.2% of the patients had distinctive features of BVN; this proportion is within the range of results reported by studies in Japan (41.9%)^25^, Korea (53.2%)^30^, and Singapore (85.2%)^18^. We classified 43.8% of PCV patients as subtype A, 10.4% of patients as subtype B, and 45.8% of patients as subtype C. We found a higher proportion of Taiwanese PCV patients that had no distinctive features of BVN shown on the ICGA images (i.e., were subtype A) compared to the Singaporean PCV population. This finding might be related to the early detection of PCV due to the routine ICGA examination for most Taiwanese nAMD patients at baseline.

Until now, classifications of PCV have been diverse. Yuzawa et al. conducted studies to elucidate the pathogenesis and origin of abnormal vessels in PCV and found that these vessels likely originated from the inner choroid. Hence, they classified PCV into two subtypes: polypoidal CNV (Type 1) and typical PCV (Type 2), according to the presence of a feeder vessel and the location of BVN in relation to Bruch’s membrane^25,26,31^; although the particularity of this classification was quite difficult to judge. Coscas et al. developed another classification, which classified PCV as idiopathic PCV and polyps associated with neovascular AMD on the basis of FA leakage, presence of BVN from ICGA, and suggestive changes in optical coherence tomography^22^. Inoue defined the PCV subtypes as PCV or P-CNV based on the presence of typical features of pachychoroid disease via optical coherent tomography angiography (OCTA), in order to discern polypoidal CNV from PCV, which is characterized by the presence of a BVN with terminating polyps^21^.

Tan et al. differentiated PCV patients into Type A (whose polyps were supplied by interconnecting channels, all without leakage by FA), Type B (who had BVN from ICGA but without leakage by FA), and Type C (who had the BVN from ICGA and leakage by FA)^17,18^. Notably, no cases with interconnecting channels (Type A) had FA leakage in their study and no etiological explanation was conclusive as to why the FA leakage seldom manifested in Type A. With the above diverse aspects of PCV subtypes, a broad range of BVN concepts needed to be adopted in clinical settings for clinical judgment. Therefore, we considered all the features of BVN described in previous literature^17,21,25,26,31^ and simplified the subtype A wherein no features of BVN were present, regardless of FA leakage.

Tan’s classification^18^, found an apparent difference in BCVA changes between Type A and Type C (13 letters vs. 6.9 letters, respectively) after six months in the EVEREST report 5^18^. However, their analysis was limited by an unbalanced treatment assignment among different types of PCV. In the current study, a similar proportion (40.0‒42.9%) of patients with each subtype of PCV received PDT. We also found a greater improvement in BCVA in subtype A compared to subtype C (8.6 letters vs. − 2.0 letters, respectively) under a 12-month treatment regime. Compared to other subtypes, subtype A in our study and Type A PCV in the EVEREST report 5 both had the best baseline BCVA^18^. Both studies also found that baseline BCVA is a significant factor associated with visual improvement. Together, these results support the idea that a better visual outcome in PCV subtype A could be related to a better baseline BCVA. It implied that a better baseline BCVA might be one of the potential factors contributing to a better treatment response which is dependent on the PCV subtypes. However, the data thus need to be interpreted with caution. A future study using OCT/OCTA to discover other factors for determining the stage of disease is needed.

The inconsistent visual outcomes in different PCV subtypes had been shown in prior studies. Using Yuzawa’s classification^25,26,31^, Jeong et al. and Nakai et al. observed a significant improvement in BCVA in Type 1 (decrease in logMAR BCVA: ~ 0.36 and 0.1) but not in Type 2 (~ 0.08 and nearly unchanged) after 3- and 12-month mono-anti-VEGF treatments, respectively^32,33^. In contrast, Honda et al. found that Type 2 may present a better BCVA improvement (estimated decrease in logMAR BCVA =  ~ 0.17) than Type 1 after PDT treatment^34^. Therefore, the effect of therapy choice is critical and should not be overlooked in evaluating the visual outcomes of different subtypes of PCV.

We observed that patients receiving combination therapy had a greater likelihood for showing improved BCVA (OR: 5.310; p = 0.0442) than those receiving the mono-anti-VEGF therapy. Our finding was consistent with the EVEREST serial studies, even though the extent of improvement with combination therapy in the EVEREST studies were numerically better. Such difference may be attributed to the difference in number of injections between studies (EVEREST: 5.2 injections for six months; current study: 3.9 injections per year)^14,16^. By contrast, the PLANET study reported that the mono-anti-VEGF therapy achieved the clinically meaningful improvement in visual acuity^35^. The different outcomes observed among studies may be related to the different treatment strategies. Patients who received mono anti-VEGF therapy in our study might be undertreated due the NHI reimbursement gap; hence, the suboptimal BCVA and CRT outcomes at 12 months. Unlike in clinical trials, the availability of anti-VEGF injection may be limited due to the reimbursement scheme and financial burden of patient self-pay in the real-world setting. Combination therapy might provide a better visual outcome if there was a limit in the available number of anti-VEGF injections in the real world.

There were some limitations to the current study. First, potential real-world variation was inherited, as is common in retrospective study design, as was the real-life variability in BCVA measurements. We did not deal with the missing data because it reflected the real-world setting. However, we applied a set of plausible values using multiple imputation to eliminate variation. Secondly, a relatively small patient pool with PCV was included because we set a criterion to enroll patients who had ICGA/FA images at baseline in order to grade PCV subtypes. Despite these factors, the current study provides a reference for the classification, treatment response, and prognosis of different subtypes of PCV. It may serve as a reference for physician’s consideration for the combination therapy and initiating treatment early for patients without distinctive features of BVN.

## Conclusion

The current study shows that combination therapy could result in better visual outcomes for PCV patients compared to mono-anti-VEGF therapy in real-life situations or when the availability of anti-VEGF injections is limited. The classification of PCV according to the presence of BVN or leakage was not associated with visual improvement. The better visual outcome in subtype A (no distinct feature of BVN) may result from better baseline BCVA. A further study using OCT/OCTA is needed. This study may serve as a reference in future real-life treatment strategies for PCV.

## Methods

### Data source

The RENOWNED and REAL studies were multicenter, open-label, single-arm, prospective, observational, non-interventional studies investigating the treatment of ranibizumab in nAMD patients (N = 202 and 228, respectively) through a one-year follow-up period^28,29^. The PCV cases were considered as a distinctive type of nAMD, the treatment for which included receiving either three reimbursed anti-VEGF injections or a self-paid combination therapy program for one year. Collected data included ICGA/FA images at baseline, BCVA, central retinal subfield thickness, presence of subretinal fluid (SRF) using optical coherence tomography (OCT), and other fundus parameters at baseline and at visits during months 3 and 12. The clinical study reports were finalized for RENOWNED^28^ in April 2015 and for REAL^29^ in December 2013.

### Study design

This *post-hoc* study analyzed data from 67 patients with PCV in the RENOWNED and REAL studies. All patients received either mono-anti-VEGF therapy or combination therapy. The National Health Insurance of Taiwan reimbursed three injections of ranibizumab per year. For combination therapy, PDT was implemented as a standard regimen (verteporfin dose 6 mg/m^2^; light dose, 50 J/cm^2^, 600 mW/cm^2^, 83 s; wavelength, 689 nm)^28,29^. Patients meeting the following criteria were enrolled: (1) PCV diagnosed by ICGA at baseline; and (2) participated at a hospital with ≥ 5 intention-to-treat (ITT) PCV cases to demonstrate a meaningful pattern of treatment choice. The ITT population was defined as patients who had received at least one injection of 0.5-mg ranibizumab (Lucentis, Novartis Pharma AG), and had data at baseline as well as at least one follow-up visit with no missing data imputation according to the original observational design. The primary endpoint was the proportion of PCV subtypes under the classification criteria defined by the study protocol. Secondary endpoints included visual and anatomical outcomes (i.e., BCVA and central retinal thickness [CRT]) of PCV patients grouped by subtypes and therapy choices.

This study was approved by the National Taiwan University Hospital Research Ethics Committee, Chang Gung Medical Foundation Institutional Review Board (IRB), Changhua Christian Hospital IRB, and Shin Kong Wu Ho Su Memorial Hospital IRB, and conducted per the applicable local regulations and the ethical principles outlined in the Declaration of Helsinki. Meanwhile, the informed consent for both study participation and further publication using unidentifying information was obtained.

### PCV subtyping

**Figure 3:**
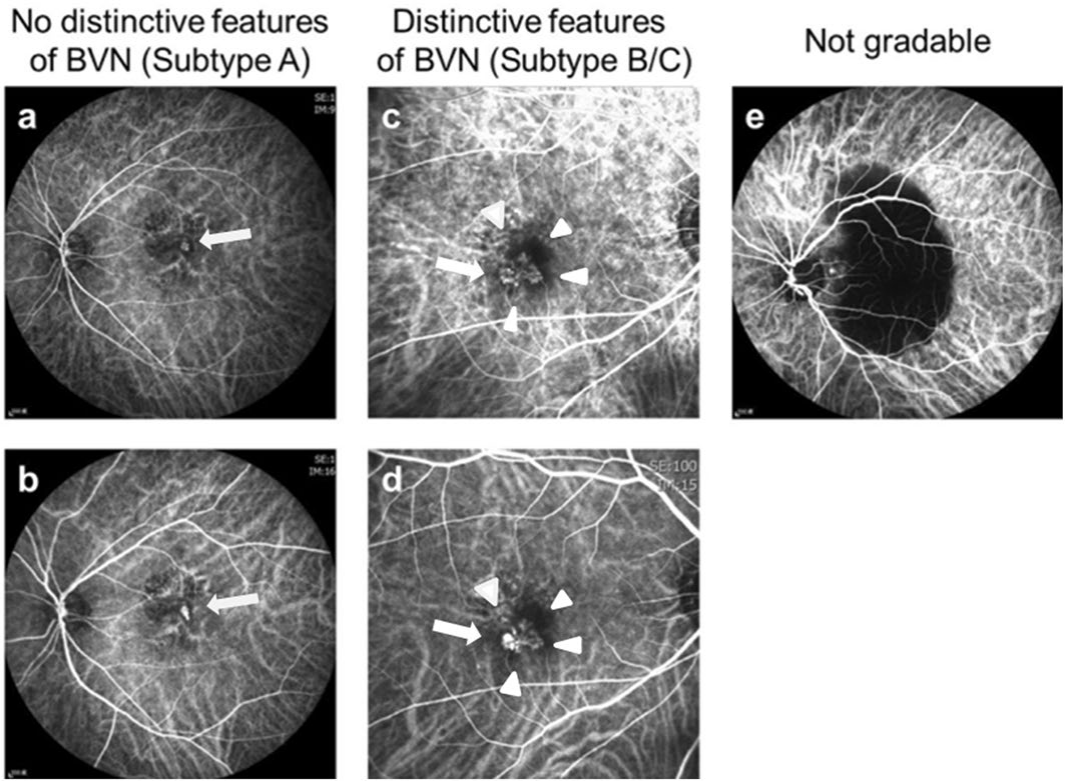
Identification of BVN for the classification of PCV subtypes using ICGA images. (**a**,**b**) ICGA images without distinct features of a branching vascular network, identified as subtype A. (**a**) Early filling of the dilated vessels (arrow). (**b**) Complete filling of the dilated vessels (arrow). (**c**,**d**) ICGA images showing branching vascular networks identified as subtype B/C (arrow head). (**c**) Early filling in distinct directions of the branching vascular network (arrow). (**d**) Complete filling of the branching vascular network (arrow). (**e**) An image that was not gradable due to a blockage caused by hemorrhage.

In this study, the definition of BVN was revised for the re-classification of PCV subtypes. Figure 3 illustrates examples of PCV patients with or without distinctive features of BVN detected using ICGA. The features of BVN included: (1) blood flow that usually radiates toward the periphery filling the vessels in distinct directions to supply the polyps (see Fig. 3c,d); (2) this filling may originate from a specific point or a feeder vessel, or from sequential filling of multiple polyps where the presence of feeder vessels can be identified^17^; and (3) early filling of the BVN could be observed within five minutes. In contrast, dilated vessels (not distinctive of BVN) consisted of fine and crisscrossing vessels without a specific point of origin or direction of flow. These vessels were usually perfused simultaneously (see Fig. 3a,b). FA was used to determine whether these abnormal vessels had leakage or not^18^. Images were classified as not-gradable if the abnormal vessels were hindered by extensive hemorrhage, high retinal pigment epithelial detachment, or poor image quality^27^.

The main readers (LY and CHY) as well as local investigators independently graded the ICGA and FA images (Heidelberg Retina Angiograph HRA and HRA 2, Germany) while anonymizing the choice of therapy and clinical course. Presence of BVN and leakage were determined. PCV was classified into three subtypes according to the following definition: Subtype A was defined as a patient who had no distinctive features of BVN by ICGA regardless of the status of FA leakage. Dilated vessels could present in this subtype. Subtype B included patients who had distinctive features of BVN by ICGA but without FA leakage; and Subtype C included patients who had distinctive features of BVN by ICGA along with FA leakage. The results of the main readers adhered to provided guided grading system and were used as the analysis dataset.

### Statistical analysis

Continuous variables were analyzed using paired t-tests or Wilcoxon signed-rank tests, and comparisons were evaluated by ANCOVA. Categorical variables were analyzed using Chi-square test or a logistic regression model with factors including PCV subtypes, therapy choices, age, and baseline BCVA.

Regarding the correlation analysis, a multiple imputation (MI) using the monotone regression method was applied to adjust for BCVA and CRT at month 12 to eliminate the potential real-world variations in the RENOWNED and REAL studies^28,29^.

A significant difference was defined as a P-value < 0.05. Data analyses were performed using statistical analysis software (SAS) version 9.4 (SAS Institute, Cary, NC, USA).

## Supplementary Information

Supplementary Information
