# Supplementary material for: Comparison of visual outcomes between therapy choices and subtypes of polypoidal choroidal vasculopathy (PCV) in Taiwan: a real-world study
Source: Sci Rep. 2021 Jan 11;11:470. doi: 10.1038/s41598-020-80731-1 (PMC7801625; doi:10.1038/s41598-020-80731-1)
Supplement: Supplementary file 1 — Supplementary Information [file 41598_2020_80731_MOESM1_ESM.docx]

**Title:**

Comparison of Visual Outcomes Between Therapy Choices and Subtypes of Polypoidal Choroidal Vasculopathy (PCV) in Taiwan: A Real-World Study

**Authors**:

Ling Yeung,^1^ Chi-Chun Lai,^2^ San-Ni Chen,^3^ Cheng-Kuo Cheng,^4^ Chung-May Yang,^6^ Yi-Ting Hsieh,^6^ Arslan Tsai,^5^ Chang-Hao Yang^6*^

^1^ Department of Ophthalmology, Chang Gung Memorial Hospital, Keelung, Keelung City, Taiwan

^2^ Department of Ophthalmology, Chang Gung Memorial Hospital, Linkou, Taoyuan City, Taiwan

^3^ Department of Ophthalmology, Changhua Christian Hospital, Changhua City, Taiwan

^4^ Department of Ophthalmology, Shin Kong Wu Ho-Su Memorial Hospital, Shilin District, Taipei City, Taiwan

^5^ Clinical Development and Medical Affairs, Novartis Taiwan

^6^ Department of Ophthalmology, National Taiwan University Hospital, Taipei, Taiwan

*Corresponding author

Supplementary Table S1. Adjusted changes in visual outcomes by therapy choice

|  | | | P-value^$^ |
| --- | --- | --- | --- |
| Covariate for BCVA changes | | |  |
| Baseline BCVA | | | 0.1504 |
| Therapy choice | | | 0.0047* |
| Covariate for CRT changes | | |  |
| Baseline CRT | | | < 0.0001* |
| Therapy choice | | | 0.0097* |
|  | n | LS mean changes ± SD at 12 months | P-value^#^ |
| BCVA changes (letters) |  |  |  |
| Combination therapy | 42 | 13.0 ± 3.80 | 0.0047* |
| Mono-anti-VEGF therapy | 24 | -1.3 ± 2.90 |  |
| CRT changes (μm) |  |  |  |
| Combination therapy | 36 | -106.2 ± 20.52 | 0.0097* |
| Mono-anti-VEGF therapy | 22 | -36.4 ± 15.26 |  |
| ^$^Type 3 test for fixed effects  ^#^ANCOVA was applied to the LS mean changes by considering the effects of baseline level and therapy choice.  *P-values < 0.05 are considered to be significant differences  Abbreviations: SD, standard deviation; VEGF, vascular endothelial growth factor; BCVA, best-corrected visual acuity; CRT, central retina thickness. | | | |

Supplementary Table S2. Adjusted visual outcomes by PCV subtypes considering real-world variations

|  | **n** | **Mean ± SD** | **P-value^$^** |
| --- | --- | --- | --- |
| BCVA (letter) |  |  |  |
| Baseline |  |  | 0.3841 |
| Subtype A | 17 | 58.9 ± 8.53 |  |
| Subtype B | 5 | 50.8 ± 11.84 |  |
| Subtype C | 21 | 55.0 ± 15.00 |  |
| Month 12 |  |  | 0.0381* |
| Subtype A | 12 | 65.0 ± 9.31 |  |
| Subtype B | 4 | 55.3 ± 12.53 |  |
| Subtype C | 14 | 52.3 ± 13.85 |  |
| CRT (μm) |  |  |  |
| Baseline |  |  | 0.6340 |
| Subtype A | 17 | 309.1 ± 69.25 |  |
| Subtype B | 5 | 275.4 ± 31.89 |  |
| Subtype C | 19 | 309.4 ± 83.72 |  |
| Month 12 |  |  | 0.8264 |
| Subtype A | 15 | 239.4 ± 52.42 |  |
| Subtype B | 4 | 251.0 ± 27.75 |  |
| Subtype C | 15 | 258.1 ± 110.78 |  |
| ^$^Type 3 test of fixed effects  *P-value < 0.05 considered as a significant difference  Abbreviations: SD, standard deviation; BCVA, best-corrected visual acuity; CRT, central retina thickness; PCV, polypoidal choroidal vasculopathy | | | |

Supplementary Table S3. Prognostic factors for BCVA and CRT of PCV patients at 12 months

|  | **Estimated** | **Standard error** | **P-value^$^** |
| --- | --- | --- | --- |
| Effects on BCVA |  |  |  |
| Baseline BCVA | 0.7029 | 0.2681 | 0.0156* |
| Number of polyps | -2.5030 | 1.8617 | 0.19255 |
| GLD of the largest polyps | -0.00039 | 0.02581 | 0.9880 |
| GLD of the whole lesion | -0.00065 | 0.00257 | 0.8024 |
| Had cluster-type polyps | 18.3834 | 9.7154 | 0.0717 |
| Had involvement of subfoveal location | 0.7172 | 8.1119 | 0.9303 |
| Effects on CRT |  |  |  |
| Baseline CRT | -0.4252 | 0.1704 | 0.0210* |
| Number of polyps | 4.7883 | 6.4402 | 0.4654 |
| GLD of the largest polyps | -0.02161 | 0.08955 | 0.8117 |
| GLD of the whole lesion | -0.01422 | 0.009443 | 0.1470 |
| Had cluster type of polyps | -30.7664 | 33.8256 | 0.3734 |
| Had involvement of subfoveal location | -86.8515 | 28.5728 | 0.0062* |
| ^$^Logistic regression was applied.  *P-values < 0.05 are considered a significant difference  Abbreviations: BCVA, best-corrected visual acuity; CRT, central retina thickness; PCV, polypoidal choroidal vasculopathy; GLD, greatest linear dimension | | | |
